# Enhancing Neurocognitive Health via Activity, Nutrition and Cognitive Exercise (ENHANCE): A Randomized Controlled Trial

**DOI:** 10.1002/jcsm.13830

**Published:** 2025-06-04

**Authors:** Li‐Ning Peng, Pei‐Lin Lee, Kun‐Hsien Chou, Wei‐Ju Lee, Ching‐Po Lin, Chih‐Wen Chang, Chih‐Kuang Liang, Chih‐Ping Chung, Fei‐Yuan Hsiao, Liang‐Kung Chen

**Affiliations:** ^1^ Center for Geriatric and Gerontology Taipei Veterans General Hospital Taipei City Taiwan; ^2^ Center for Healthy Longevity and Aging Sciences National Yang Ming Chiao Tung University Taipei City Taiwan; ^3^ Institute of Neuroscience National Yang Ming Chiao Tung University Taipei City Taiwan; ^4^ Brain Research Center National Yang Ming Chiao Tung University Taipei City Taiwan; ^5^ Department of Family Medicine Taipei Veterans General Hospital Yuanshan Branch Yilan County Taiwan; ^6^ Department of Education and Research Taipei City Hospital Taipei City Taiwan; ^7^ Center for Geriatrics and Gerontology Kaohsiung Veterans General Hospital Kaohsiung City Taiwan; ^8^ Division of Neurology, Department of Internal Medicine Kaohsiung Veterans General Hospital Kaohsiung City Taiwan; ^9^ Department of Geriatric Medicine, School of Medicine National Yang Ming Chiao Tung University Taipei Taiwan; ^10^ Department of Neurology, Neurological Institute Taipei Veterans General Hospital Taipei City Taiwan; ^11^ Graduate Institute of Clinical Pharmacy, College of Medicine National Taiwan University Taipei Taiwan; ^12^ School of Pharmacy, College of Medicine National Taiwan University Taipei Taiwan; ^13^ Department of Pharmacy National Taiwan University Hospital Taipei Taiwan; ^14^ Taipei Municipal Gan‐Dau Hospital (Managed by Taipei Veterans General Hospital) Taipei City Taiwan

**Keywords:** brain structure, multidomain intervention, neuroplasticity, older adults, urban–rural disparities

## Abstract

**Background:**

While multidomain interventions show promise for promoting healthy aging, their impact on brain structure remains unclear. This randomized controlled trial (ENHANCE) assessed the efficacy of a 12‐month group‐based multidomain intervention on brain structure and function in community‐dwelling older adults, with particular attention to urban–rural disparities.

**Methods:**

The ENHANCE trial delivered twice‐weekly group‐based multidomain sessions (physical exercise, cognitive training and nutrition education) in urban and rural communities for 12 months, while the control group received quarterly telephone education. A total of 88 participants completed the trial (attendance rates > 60% across all sites), with 76 completing longitudinal MRI assessments (intervention: *n* = 35; control: *n* = 41). The intervention group (*n* = 44; 75.0% female) was significantly older than the control group (*n* = 44; 70.5% female) (75.0 ± 6.6 vs. 72.3 ± 5.0 years, *p* = 0.035) and had lower BMI (23.4 vs. 25.2 kg/m^2^, *p* = 0.016) at baseline. The primary outcomes were brain structures (voxel‐based changes in brain grey matter volume [GMV]), while secondary outcomes were functional outcomes (physical performance, nutritional status, cognitive function, psychosocial assessments and cardiometabolic biomarkers).

**Results:**

Using two‐stage tensor‐based morphometry analysis, the intervention group demonstrated significantly less GMV reduction in regions over the left inferior temporal lobe compared with controls over 12 months (*p* < 0.05). Using generalized estimating equation (GEE) model, the intervention group showed enhanced physical performance at 6 months (5‐time chair rise test: −1.19 s; 95% CI, −2.24 to −0.13; *p* = 0.028) and improved cognitive function by 12 months (Montreal Cognitive Assessment: +1.32 points; 95% CI, 0.10–2.54; *p* = 0.034). Cardiometabolic improvements included increased HDL‐C (+6.65 mg/dL, *p* < 0.001) and decreased triglycerides (−16.07 mg/dL, *p* = 0.025) at 12 months in GEE models. In subgroup analyses, rural participants showed preserved GMV in additional regions including the cerebellum (Crus I and II) and occipital cortex with greater cognitive improvements (MoCA: +3.06 points; 95% CI, 0.84–5.27; *p* = 0.007), while urban participants showed greater GMV reduction in the left temporal‐occipital fusiform cortex but achieved superior physical performance gains (5‐time chair rise test: −1.85 s; 95% CI, −3.07 to −0.64; *p* = 0.003).

**Conclusions:**

This study demonstrates that multidomain interventions can induce neuroplasticity in older adults, with differential effects on brain structure and function between urban and rural participants, emphasizing the need for tailored approaches that consider sociocultural factors to optimize healthy aging across diverse populations.

## 1 Introduction

Population aging presents unprecedented global challenges [1], prompting the World Health Organization to emphasize functional ability maintenance for healthy aging. In this context, enhancing brain health has emerged as a priority to optimize late‐life well‐being [2], further supported by robust evidence linking neuroanatomical alterations, modifiable risk factors and cognitive reserve [3]. Consequently, multidomain interventions incorporating physical exercise, cognitive training and lifestyle modifications have demonstrated promising results in promoting physical and cognitive functions, leading to the establishment of the World‐Wide FINGERS Network [4, 5, 6, 7, 8, 9, 10] for coordinating global implementation. Despite these advances, observed heterogeneity in intervention effects underscores the need to investigate various contributing factors, particularly participant characteristics and socio‐cultural disparities.

Building on these developments, our previous trials (THISCE and TIGER) [4, 10] demonstrated improvements in physical health, mental well‐being and chronic disease management, although intervention effects declined with reduced group session frequency. Currently, two critical knowledge gaps persist in multidomain intervention research: insufficient neuroimaging evidence of brain structural changes, as exemplified by FINGER's inconclusive MRI findings [11], and limited understanding of urban–rural implementation disparities. Notably, while some studies included brain magnetic resonance imaging (MRI) assessments, none have made MRI changes a primary outcome, thus leaving the brain mechanisms underlying cognitive improvements unclear.

Motivated by the existing knowledge gap, we designed and conducted this randomized controlled trial (RCT). This trial primarily aimed to investigate brain structural changes (voxel‐based changes in brain grey matter volume [GMV]), while comprehensively assessing functional outcomes (physical performance, cognitive function, psychosocial assessments and cardiometabolic biomarkers) in urban and rural older adults. By implementing an enhanced intervention frequency compared with previous trials (THISCE and TIGER), this study also addressed existing knowledge gaps in intervention‐induced brain structural changes. The advanced voxel‐based MRI analytical approach provided superior sensitivity in detecting subtle volumetric brain changes, offering precise evaluation of structural neuroplasticity.

## 2 Methods

### 2.1 Study Participants

Trained staff conducted recruitment drives between 8 August 2021 and 30 June 2022 by visiting community centres to invite residents for participation and screen their eligibility through interviews. Inclusion criteria targeted community‐dwelling older adults (≥ 65 years) residing in Taipei City (urban) or Yi‐Lan County (rural), Taiwan. Participants were selected based on potential risk factors for dementia and disability, including slow gait speed (< 1 m/s on the 6‐m walk test), weakness (dominant handgrip strength < 28 kg for men and < 18 kg for women) and subjective cognitive declines [12, 13, 14, 15, 16]. All study participants met eligibility criteria, presenting with no cognitive impairment, physical disabilities or acute conditions that could affect functional status. The inclusion and exclusion criteria aligned with established protocols from comparable clinical trials, including MAPT [7], THISCE [4] and TIGER [10]. Exclusion criteria included a prior diagnosis of dementia or other neurological disorders (stroke or Parkinsonism) confirmed by geriatrician or neurologist, self‐reported or caregiver‐reported total/partial dependence for activities of daily living (ADLs), anticipated life expectancy less than 6 months due to active illness, severe hearing or visual impairment, major depression/anxiety or other significant illnesses potentially affecting compliance. Additionally, participants were excluded if they were institutionalized, or currently enrolled in other clinical trials or research.

Eligible participants were randomly assigned to either the multidomain interventions group or the usual care group in a 1:1 ratio. Randomization was performed using a computer‐generated random number sequence created with Excel 2017 (Microsoft, Redmond, WA, USA). The random allocation codes were placed in an opaque bag, and participants drew their allocation randomly, ensuring unbiased group assignment. Neither the recruitment staff nor the participants were able to see the allocation codes while drawing. In addition, outcome assessors were blinded to group allocation throughout the intervention period and during all outcome assessments. Data analysts were also blinded to group assignment during statistical analysis through the use of coded identifiers that did not reveal intervention status. These approaches help to reduce potential bias in outcome assessment and improve the internal validity of the study.

A sample size of 45 participants per group was calculated using G*Power 3.1.9.7 based on the established association between slow gait speed and cognitive decline [15]. Accounting for a 10% withdrawal rate, a sample size of 50 participants per group was determined to be sufficient to detect a moderate‐to‐large effect size at an alpha level of 0.1 with 80% statistical power.

### 2.2 Ethical Compliance and Trial Registration

The research protocol was approved by the Institutional Review Board of National Yang Ming Chiao Tung University (YM109161F) and adhered to the 1964 Declaration of Helsinki and national regulations. All participants provided written informed consent prior to participation. The trial has been registered at the ClinicalTrials.gov (NCT05828043).

### 2.3 Intervention Programmes

The intervention in the ENHANCE trial mirrored that of prior community‐based RCTs (THISCE and TIGER) [4, 10] by incorporating physical exercise, cognitive training, nutritional counselling, social networking and chronic condition management education. However, ENHANCE maintained the group‐based format twice weekly for 12 months, unlike the gradual shift to home‐based interventions in THISCE [4] and TIGER [10]. The intervention sessions consisted of three components: (a) 15 min of nutritional counselling aligned with national dietary guidelines for older adults, (b) 1 h of cognitive training focusing on reasoning and memory exercises and (c) 45 min of physical exercises targeting muscle strength, balance, flexibility and endurance. The ENHANCE trial intervention sessions were delivered across four community centres in Taipei City (urban) and Yi‐Lan County (rural). Conversely, participants in the control group received standardized telephone‐based health education interventions. These follow‐up calls simply monitored their health status and provided education on exercise and nutritional guidelines established by the Administration of Health Promotion, Ministry of Health and Welfare.

### 2.4 Demographic Characteristics

Baseline demographics (age, sex, education year, current smoking status, current drinking status and income), self‐reported chronic conditions (hypertension, coronary heart disease, cerebrovascular disease, diabetes mellitus (DM), chronic obstructive pulmonary disease (COPD), chronic kidney disease, osteoporosis, osteoarthritis, depression and cancer were recorded. Charlson Comorbidity Index (CCI) was used to estimate the overall disease burden of the participants. Cardiovascular risk factors were assessed using a combination of objective measurements and self‐reported diagnoses in this study.

### 2.5 Primary Outcomes: Brain Structures

**FIGURE 1:**
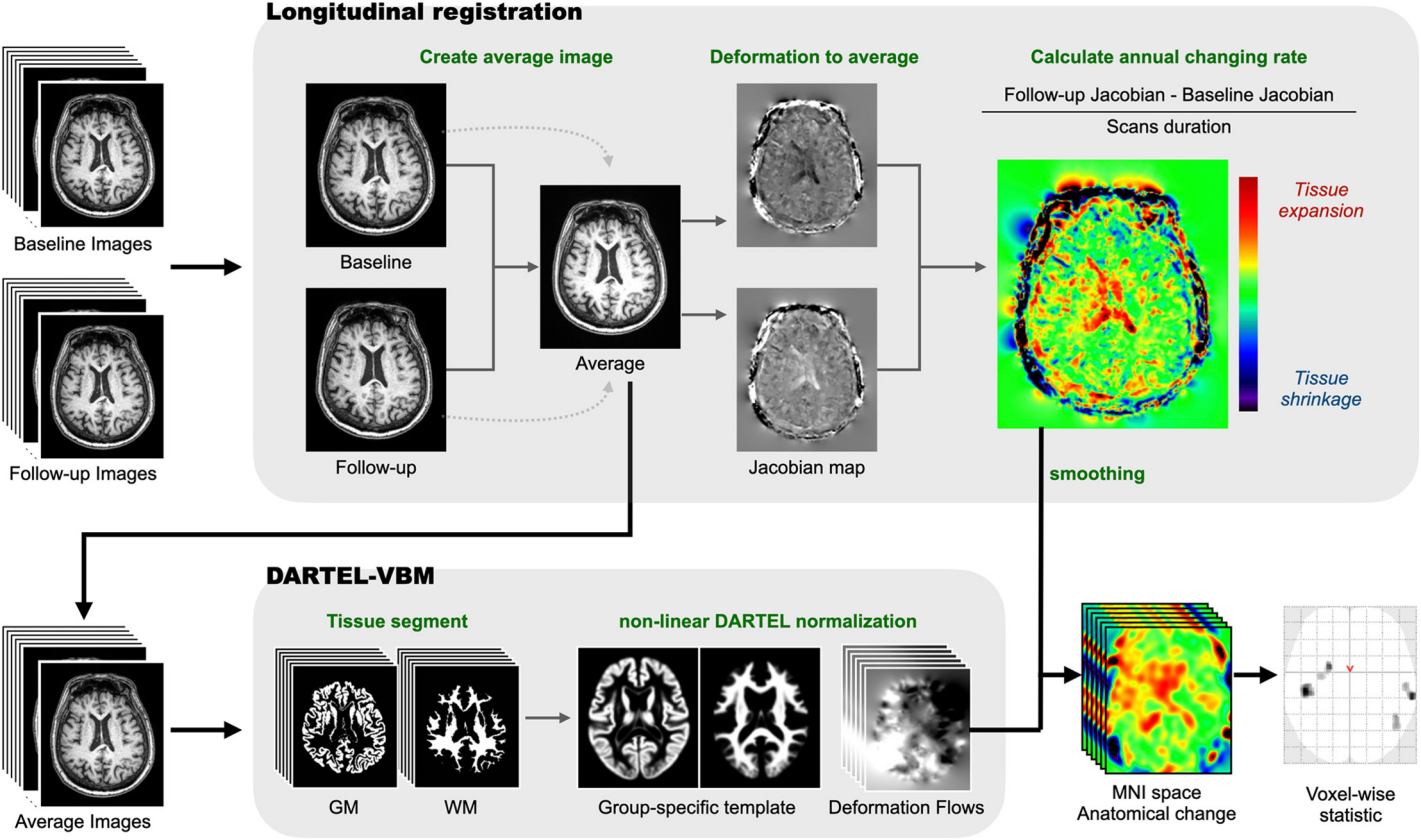
Neuroimage processing: two‐stage tensor‐based morphometry. A longitudinal registration of the baseline and follow‐up T1‐weighted images for each participant was performed (upper part). This process employing an inverse‐consistent non‐linear registration approach calculated the mid‐point average images and the corresponding voxel‐wise Jacobians determinants. These determinants were used to calculate the rate of brain changes over time, where warmer colours (positive values) denoted volume expansion and cooler colours (negative values) represented volume reduction. Subsequent standard voxel‐based morphometry (VBM) was applied to these average images (lower part), segmenting them into different tissue types and utilizing this segmentation to create group‐specific templates via a fast diffeomorphic registration algorithm. The deformation fields were applied to the individual Jacobian rate maps, which were then transformed into standard Montreal Neurological Institute (MNI) space and smoothed for voxel‐wise statistical analysis of brain expansion or contraction rates.

The brain structures were assessed by evaluating the changes in GMV across all voxels in the entire brain, using MRI data collected from baseline to the 12 months follow‐up (Figure 1). A two‐stage tensor‐based morphometry methodology was applied using the statistical parametric mapping software (SPM12, Version 7771, Wellcome Institute of Neurology, University College London, accessible at https://www.fil.ion.ucl.ac.uk/spm/software/spm12/) within the MATLAB environment (Version R2023a, Mathworks, Natick, MA). This approach is integral for the precise estimation of voxel‐wise morphometric alterations over time [17, 18, 19]. A detailed description of the brain MRI acquisition and analytic methods is provided in the Supporting Information.

### 2.6 Secondary Outcomes: Functional Outcomes

Physical performance, including handgrip strength, walking speed, 5‐time chair rise test and body composition, was assessed at baseline, 6 months and 12 months. Handgrip strength was assessed using a digital dynamometer (Smedley's Dynamo Meter; TTM, Tokyo, Japan) on the dominant hand. Three trials of handgrip strength were conducted, and the best result was recorded for analysis. The 6‐m usual gait speed was evaluated with a static start and no deceleration was measured. The 5‐time chair rise test evaluates lower extremity strength, balance and mobility by measuring the time needed to stand up and sit down five times from a chair. Body composition including body muscle mass, body fat mass, skeletal muscle mass and total body fat (%) was quantified using the bioelectrical impedance analysis (BIA) (Inbody S10, Seoul, South Korea). Appendicular skeletal muscle mass (ASM) was defined as the sum of lean body mass from both arms and legs, adjusted for participant height (in metres) squared (ht^2^) as the relative ASM (RASM) for analysis. The Clinical Frailty Scale (CFS) was used to evaluate frailty.

The nutritional status of participants was evaluated using the Mini Nutritional Assessment (MNA) tool at baseline and follow‐up time points. The MNA is a validated screening and assessment instrument with scores ranging from 0 to 30.

Cognitive function was evaluated using a Chinese version of the Montreal Cognitive Assessment (MoCA) screening tool [20]. The full MoCA battery covers domains including visuospatial executive, naming, concentration, language, abstract thinking, delayed recall and orientation. Psychosocial assessments included psychological function, personal mastery and social frailty. Psychological function was measured by the Center for Epidemiologic Studies Depression (CES‐D) questionnaire [21], which consists of 20 items that evaluate various aspects of depressive symptoms, including depressed mood, feelings of guilt and worthlessness, psychomotor retardation, loss of appetite and sleep disturbances. Participants rate the frequency of experiencing each symptom over the past week on a 4‐point Likert scale ranging from 0 (*rarely or none of the time*) to 3 (*most or all of the time*). Total scores range from 0 to 60, with higher scores indicating greater depressive symptoms. A cut‐off score of 16 or higher is typically used to identify individuals at risk for clinical depression. Personal mastery, a surrogate marker of resilience, was measured using Pearlin mastery scale [22, 23], which consisted of 7 items as the following: (1) I have little control over the things that happen to me, (2) what happens to me in the future mostly depends on me, (3) there is really no way I can solve some of the problem I have, (4) there is little I can do to change many of the important things in my life, (5) I can do just about anything I really set my mind to, (6) I often feel helpless in dealing with life's problems, (7) sometimes I feel that I'm being pushed around in life. The total score for the individual participant was summed by the 7 items, ranging from 7 to 28 with a higher score indicating a higher level of personal mastery. Moreover, this study used social frailty to represent the social networking and social isolation of the study participants, which was evaluated by 8 items that evaluate various aspects of social frailty [24, 25].

For cardiometabolic biomarkers, peripheral venous blood samples were obtained from all participants after a 10‐h overnight fast, and various cardiometabolic parameters were measured, including total cholesterol, low‐density lipoprotein cholesterol (LDL‐C), high‐density lipoprotein cholesterol (HDL‐C), triglyceride, fasting glucose and HgbA1c.

### 2.7 Statistical Analyses of Primary Outcomes

Neuroimaging statistical analysis was performed using SPM12. We constructed a general linear model to perform whole‐brain voxel‐wise statistical analysis. This analysis employed a single‐factor‐two‐level (intervention vs. control groups) analysis of covariance model (ANCOVA). Several identified factors were included as nuisance covariates to account for potential confounding factors. This approach was instrumental in identifying between‐group differences in the GMV changes of any voxel from baseline to 12 months throughout the whole brain. To account for the multiple comparisons issue, the resulting statistical maps were thresholded at voxel‐level *p*‐value of 0.005, with a cluster‐level extent of 237 voxels. This thresholding was based on family‐wise error correction at an alpha value threshold of 0.05, ensuring that our findings were robust and statistically significant. In addition, for data reusability and transparency, all the unthresholded voxel‐wise statistical maps have been made available at the NeuroVault website (https://neurovault.org/collections/16106/).

### 2.8 Statistical Analyses of Secondary Outcomes

All analyses followed a per‐protocol approach rather than intention‐to‐treat, as the study's focus on neuroanatomical changes required complete longitudinal data for meaningful interpretation. This analytical strategy was chosen to align with the primary outcome measurement requirements, where paired imaging data was essential for calculating individual‐level volume changes between time points. While this approach may impact the generalizability of findings, baseline characteristics remained comparable between groups in the final analysis sample, suggesting the randomization integrity was maintained. Numerical data were presented as mean values accompanied by standard deviations (SDs), while categorical data were reported as frequencies along with percentages.

Baseline characteristics between groups were compared using statistical tests such as the Student's *t*‐test for numerical variables and the chi‐square test for categorical variables when appropriate. A generalized estimating equation (GEE) model was employed to assess the intervention effects on outcomes of interest. As the recruitment of study participants was conducted in two different sites (i.e., Taipei and Yi‐Lan), a pre‐planned sub‐cohort analysis was conducted to see whether the intervention effects might be different in different sites. Statistical significance in all analyses was determined by a two‐tailed *p*‐value of less than 0.05. The statistical analysis was conducted using IBM SPSS Statistics for Mac OS Version 26 (IBM Corp., Armonk, NY, USA) and SAS statistical package, Version 9.4 (SAS Institute, Inc., Cary, NC, USA).

## 3 Results

### 3.1 Demographics

**FIGURE 2:**
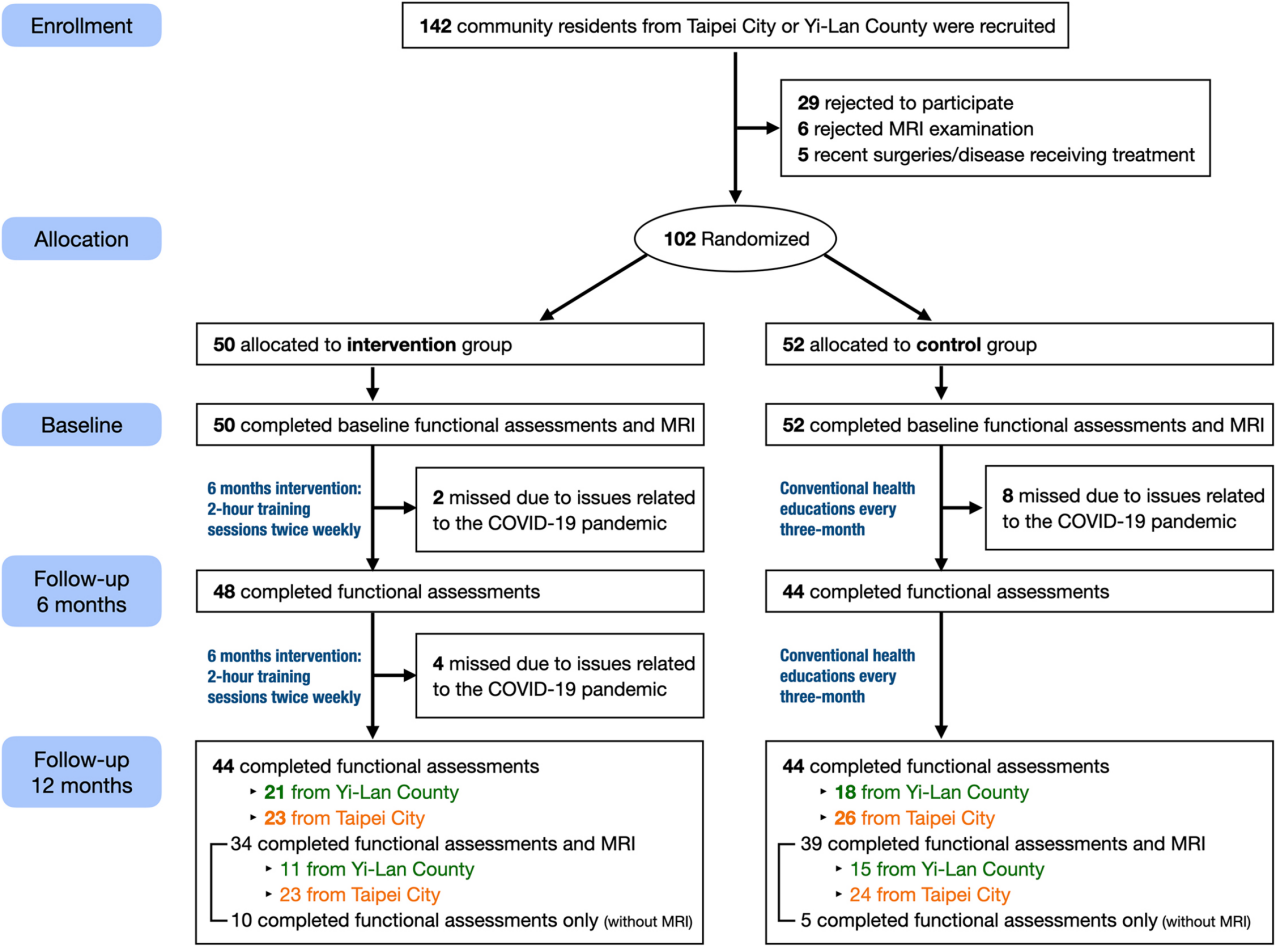
Study recruitment algorithm.

Since 1 November 2021, a total of 142 community‐dwelling older people have been screened for study (Figure 2). Eventually, a total of 102 participants were initially enrolled in the study, including 50 in the intervention group and 52 in the control group. The first assessment took place on 1 November 2021, and all cases completed the 12‐month assessment by 19 June 2023. The final analyses included 88 participants who had completed both the 6 and 12 months of functional assessments, evenly divided between intervention (*n* = 44) and control (*n* = 44) groups. The intervention sessions were conducted at two urban sites in Taipei (attendance rates: 73.31% and 87.34%) and two rural sites in Yi‐Lan (attendance rates: 85.59% and 63.33%). The attendance rates exceeded 60% across all sites, and no intervention‐related adverse events were reported throughout the study period.

The groups were comparable in sex distribution (75.0% vs. 70.5% female, *p* = 0.811), education levels (70.5% with ≥ 9 years in both groups, *p* = 1.000) and site distribution (*p* = 0.668). Both groups had similar rates of hypertension (54.5% vs. 50.0%, *p* = 0.871) and other comorbidities. The mean CCI was similar between groups (0.8 vs. 1.0, *p* = 0.532). However, the intervention group was significantly older (75.0 vs. 72.3 years, *p* = 0.035) and had a lower body mass index (BMI) (23.4 vs. 25.2 kg/m^2^, *p* = 0.016) compared with the control. Besides, a notable difference in the prevalence of DM between groups was noted (15.9% in intervention vs. 36.4% in control, *p* = 0.051) (Table 1).

**TABLE 1:** Demographics of study participants.

	Total population				Yi‐Lan cohort				Taipei cohort			
	Total (*n* = 88)	Intervention (*n* = 44)	Control (*n* = 44)	*p*‐value	Total (*n* = 39)	Intervention (*n* = 21)	Control (*n* = 18)	*p*‐value	Total (*n* = 49)	Intervention (*n* = 23)	Control (*n* = 26)	*p*‐value
Site, *n* (%)				0.668								
Yi‐Lan	39 (44.3%)	21 (47.7%)	18 (40.9%)									
Taipei	49 (55.7%)	23 (52.3%)	26 (59.1%)									
Age (years), mean (SD)	73.6 (6.0)	75.0 (6.6)	72.3 (5.0)	0.035	74.0 (7.3)	74.7 (8.2)	73.3 (6.3)	0.545	73.3 (4.6)	75.2 (4.9)	71.7 (3.7)	0.006
Sex, *n* (%)				0.811				0.432				1.000
Male	24 (27.3%)	11 (25.0%)	13 (29.6%)		8 (20.5%)	3 (14.3%)	5 (27.8%)		16 (32.7%)	8 (34.8%)	8 (30.8%)	
Female	64 (72.7%)	33 (75.0%)	31 (70.5%)		31 (79.5%)	18 (85.7%)	13 (72.2%)		33 (67.4%)	15 (65.2%)	18 (69.2%)	
Education year, *n* (%)				0.100				1.000				0.612
< 9	26 (29.5%)	13 (29.5%)	13 (29.5%)		17 (43.6%)	9 (42.9%)	8 (40.4%)		4 (8.2%)	1 (4.4%)	3 (11.5%)	
≥ 9	62 (70.5%)	31 (70.5%)	31 (70.5%)		22 (56.4%)	12 (57.1%)	10 (55.6%)		45 (91.8%)	22 (95.6%)	23 (88.5%)	
Current smoker, *n* (%)	4 (4.5%)	2 (4.5%)	2 (4.5%)	0.568	4 (10.3%)	2 (9.5%)	2 (11.1%)	0.285	11 (22.5%)	4 (17.4%)	7 (26.8%)	0.666
Current drinking, *n* (%)	34 (38.6%)	19 (43.2%)	15 (34.1%)	0.594	9 (33.1%)	7 (33.4%)	2 (11.1%)	0.782	35 (71.4%)	16 (69.6%)	19 (73.1%)	0.776
Income (TWD), *n* (%)				0.135				0.089				0.420
< 800 000	68 (77.3%)	38 (88.9%)	30 (68.2%)		36 (92.3%)	21 (100%)	15 (83.3%)		32 (65.3%)	17 (73.9%)	15 (57.7%)	
≥ 800 000	15 (17.1%)	5 (8.2%)	10 (22.7%)		3 (7.7%)	0 (0.0%)	3 (16.7%)		12 (24.5%)	5 (21.7%)	7 (26.9%)	
Unknown	5 (5.6%)	1 (2.2%)	4 (9.1%)		0 (0.0%)	0 (0.0%)	0 (0.0%)		5 (10.2%)	1 (4.4%)	4 (15.4%)	
CCI, mean (SD)	0.9 (1.2)	0.8 (1.1)	1.0 (1.2)	0.532	0.4 (0.7)	0.4 (0.8)	0.4 (0.5)	0.943	1.3 (1.4)	1.2 (1.2)	1.3 (1.5)	0.664
Comorbid conditions												
Hypertension	46 (52.3%)	24 (54.5%)	22 (50.0%)	0.871	23 (59.0%)	16 (76.2%)	7 (38.9%)	0.025	23 (46.9%)	8 (34.8%)	15 (57.7%)	0.154
Coronary heart disease	15 (17.1%)	8 (18.2%)	7 (15.9%)	1.000	8 (20.5%)	5 (23.8%)	3 (16.7%)	0.702	7 (14.3%)	3 (13.0%)	4 (14.4%)	1.000
Cerebrovascular disease	5 (5.7%)	2 (4.6%)	3 (6.8%)	1.000	0 (0.0%)	0 (0.0%)	0 (0.0%)	—	5 (10.2%)	2 (8.7%)	3 (11.5%)	1.000
Diabetes mellitus	23 (26.1%)	7 (15.9%)	16 (36.4%)	0.051	11 (28.2%)	4 (19.1%)	7 (38.9%)	0.285	12 (24.5%)	3 (13.0%)	9 (34.6%)	0.104
COPD	11 (12.5%)	5 (11.4%)	6 (13.6%)	1.000	3 (7.7%)	1 (4.8%)	2 (11.1%)	0.587	8 (16.3%)	4 (17.4%)	4 (15.4%)	1.000
Chronic kidney disease	5 (5.7%)	2 (4.6%)	3 (6.8%)	1.000	1 (2.6%)	0 (0.0%)	1 (5.6%)	0.462	4 (8.2%)	2 (8.7%)	2 (7.7%)	1.000
Osteoporosis	15 (17.1%)	7 (15.6%)	8 (18.2%)	1.000	4 (10.3%)	2 (9.5%)	2 (11.1%)	1.000	11 (22.5%)	5 (21.7%)	4 (23.1%)	1.000
Osteoarthritis	16 (18.2%)	6 (13.3%)	10 (22.7%)	0.408	1 (2.6%)	1 (4.8%)	0 (0.0%)	1.000	15 (30.6%)	5 (21.7%)	10 (38.5%)	0.233
Depression	6 (6.8%)	3 (6.7%)	3 (6.8%)	1.000	2 (5.1%)	2 (9.5%)	0 (0.0%)	0.490	4 (8.2%)	1 (4.4%)	3 (11.5%)	0.612
Cancer	11 (12.5%)	6 (13.6%)	5 (11.4%)	1.000	1 (2.6%)	1 (4.8%)	0 (0.0%)	1.000	10 (20.4%)	5 (21.7%)	5 (19.2%)	1.000
Anthropometric measurements												
Height (cm), mean (SD)	157.3 (7.8)	157.5 (7.8)	157.1 (7.9)	0.799	156.3 (7.8)	155.7 (7.9)	157.1 (7.9)	0.569	158.1 (7.8)	159.2 (7.5)	157.1 (8.0)	0.340
Weight (kg), mean (SD)	60.2 (10.1)	58.2 (8.9)	62.3 (11.0)	0.064	60.5 (7.3)	58.0 (10.1)	63.3 (12.2)	0.149	60.0 (9.3)	58.4 (7.8)	61.5 (10.3)	0.248
BMI (kg/m^2^), mean (SD)	24.3 (3.6)	23.4 (2.8)	25.2 (4.0)	0.016	24.7 (4.0)	23.8 (3.3)	25.6 (4.6)	0.156	24.0 (3.2)	23.0 (2.3)	24.9 (3.6)	0.030

Abbreviations: BMI, body mass index; CCI, Charlson comorbidity index; COPD, chronic obstructive pulmonary disease.

We also compared intervention and control groups across sites and time points (Table 1). At baseline, the groups were similar in most aspects, but some significant differences emerged. In the Taipei (urban) cohort, the intervention group was significantly older (75.2 vs. 71.7 years, *p* = 0.006) and had a lower BMI (23.0 vs. 24.9 kg/m^2^, *p* = 0.030) compared with the control group. In the Yi‐Lan (rural) cohort, the intervention group had a significantly higher prevalence of hypertension (76.2% vs. 38.9%, *p* = 0.025).

Due to the impacts of COVID‐19 pandemic, 34 out of the initial 50 participants in the intervention group and 39 out of the initial 52 participants in the control group were able to have their 12 months follow‐up brain MRI scans (Figure 2). The groups who received follow‐up brain MRI scans were comparable in sex distribution (76.5% vs. 70.5% female, *p* = 0.791), education levels and site distribution (*p* = 0.631). However, the intervention group was significantly older (75.0 vs. 71.9 years, *p* = 0.005) and had a lower BMI (23.2 vs. 25.4 kg/m^2^, *p* = 0.008) compared with the control group (Table S1). We also compared intervention and control groups across sites and time points (Table S1).

### 3.2 Neuroimaging Outcomes

Longitudinal MRI datasets were acquired from 35 participants in the intervention group and 41 in the control group. The two‐stage tensor‐based morphometry analytical framework was employed to extract individual‐level changes in volume between subsequent brain scans over time (referred to as volume change rate). This method mapped voxel‐wise volume change rates across the entire brain (Figure 1). Subsequently, the volume change maps of the intervention group were compared with those of the control group.

**FIGURE 3:**
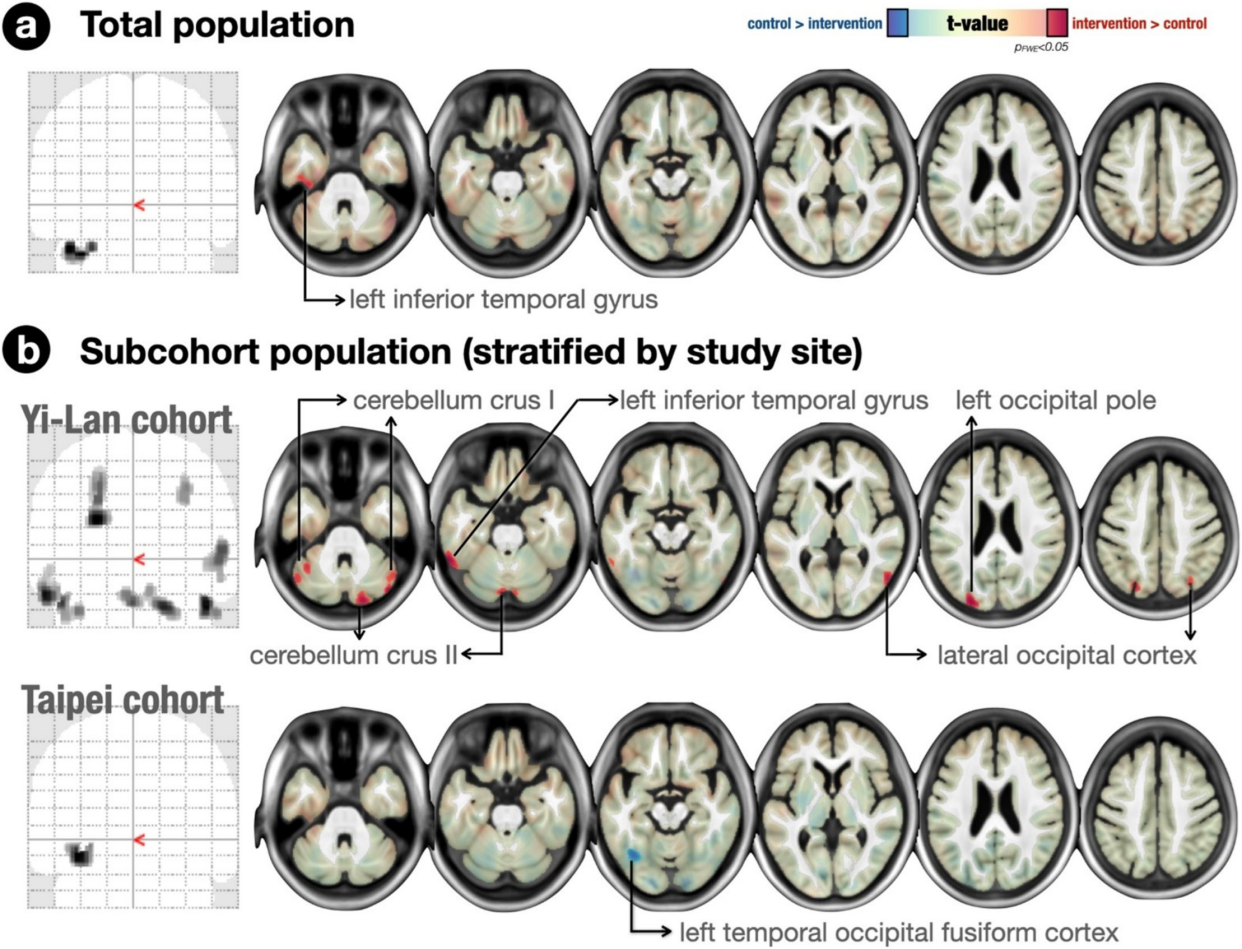
Differences of neuroanatomic changes between intervention and control groups. Grey matter volume (GMV) change rate differences between the intervention and control groups after 12 months, adjusted for study site, age, years of education and body mass index (BMI), were discovered in regions over the left inferior temporal lobe (left inferior temporal gyrus) (top row). When stratified by site, the Yilan subcohort analysis showed that, in addition to the left inferior temporal gyrus, the intervention group exhibited less GMV reduction or even expansion in regions such as the cerebellum (Crus I and II) and the occipital cortex (left occipital pole and bilateral lateral occipital cortex) after similar adjustments (middle row). In contrast, the Taipei subcohort demonstrated greater GMV reduction in the left temporal‐occipital fusiform cortex in the intervention group (bottom row). Hot colours represent areas with significantly increased GMV change, while cold colours indicate decreased GMV change in the intervention group compared with the control group.

Baseline comparisons indicated that there were no pre‐existing differences in brain volume changes between the groups (Table S1). However, significant group differences in regions over the left inferior temporal lobe (left inferior temporal gyrus) were observed after adjusting for age, site, education years and BMI. (Figure 3). The results showed that the intervention group had less volume reduction or a volume increment in regions over the left inferior temporal lobe (left inferior temporal gyrus) than the control group over 12 months.

We then stratified the study population by study sites (Figure 3). In the Yi‐Lan (rural) sub‐cohort, the analyses indicated that, beyond regions over the left inferior temporal lobe (left inferior temporal gyrus), the intervention group exhibited less volume reduction or even volume increment in regions such as the cerebellum (Crus I and II) and the occipital cortex (left occipital pole and bilateral lateral occipital cortex), after adjusting for age, years of education and BMI. Conversely, in the Taipei (urban) sub‐cohort, the intervention group showed greater volume reduction in the left temporal‐occipital fusiform cortex. Detailed numerical values for the neuroimaging results are presented in Table S2.

### 3.3 Physical and Cognitive Functional, Psychosocial and Cardiometabolic Outcomes

Regarding intervention effects on clinical outcomes, several significant changes were observed in the GEE model. At 6th month, the intervention group showed improvement in the 5‐time chair rise test (−1.19 s, *p* = 0.028) compared with the controls, after adjustment for site, age and BMI (Table 2).

**TABLE 2:** Intervention effect on physical, psychosocial, cognitive and cardiometabolic outcomes.

Outcome	Intervention (*n* = 44)			Control (*n* = 44)			Group comparison at 6 M (vs. baseline)			Group comparison at 12 M (vs. 6 M)		
	Baseline	6 M	12 M	Baseline	6 M	12 M	Difference in group means (95% CI)	Beta (SE)	*p*‐value	Difference in group means (95% CI)	Beta (SE)	*p*‐value
Physical performance												
5‐time chair rise test (s)	10.1 (2.8)	9.2 (2.7)	8.7 (2.2)	9.8 (3.6)	10.1 (3.7)	9.5 (3.2)	−1.19 (−2.24 to −0.13)	−1.19 (0.54)	0.028	0.13 (−0.82 to 1.08)	0.13 (0.48)	0.792
6‐m walk (s)	1.1 (0.3)	1.2 (0.3)	1.2 (0.2)	1.1 (0.3)	1.2 (0.3)	1.2 (0.2)	0.01 (−0.09 to 0.12)	0.01 (0.05)	0.807	0.02 (−0.06 to 0.11)	0.02 (0.04)	0.601
Hand grip strength (kg)	21.9 (7.3)	22.6 (7.6)	22.8 (7.4)	22.4 (6.4)	22.9 (6.9)	23.3 (6.9)	0.13 (−1.38 to 1.64)	0.13 (0.77)	0.866	−0.21 (−1.34 to 0.93)	−0.21 (0.58)	0.720
Frailty (CFS)	2.5 (0.6)	2.4 (0.7)	2.5 (0.6)	2.5 (0.7)	2.5 (0.6)	2.4 (0.7)	−0.16 (−0.34 to 0.02)	−0.16 (0.09)	0.0842	0.27 (0.10 to 0.44)	0.27 (0.09)	0.001
Body composition												
Body muscle mass (kg)	21.2 (4.4)	21.1 (4.0)	20.4 (4.1)	22.3 (4.7)	21.6 (4.3)	21.9 (4.0)	0.47 (−0.21 to 1.12)	0.47 (0.34)	0.180	−0.91 (−1.35 to −0.47)	−0.91 (0.23)	< 0.001
Body fat mass (kg)	18.8 (4.9)	18.0 (4.9)	19.4 (4.5)	21.0 (7.0)	21.4 (7.3)	21.3 (7.0)	−1.20 (−2.30 to 0.21)	−1.20 (0.71)	0.096	1.48 (0.44–2.52)	1.48 (0.53)	0.005
Skeletal muscle mass (kg)	2.4 (0.4)	2.4 (0.3)	2.4 (0.3)	2.9 (3.1)	2.3 (0.4)	2.4 (0.4)	0.58 (−0.34 to 1.50)	0.58 (0.47)	0.215	−0.13 (−0.22 to −0.03)	−0.13 (0.05)	0.008
Total body fat (%)	32.1 (6.1)	31.1 (5.6)	33.5 (5.3)	33.4 (8.2)	33.4 (8.8)	34.0 (7.6)	−0.99 (−3.07 to 1.10)	−0.99 (1.06)	0.353	1.77 (0.08–3.46)	1.77 (0.86)	0.041
RASM (kg/m^2^)	6.4 (1.1)	6.3 (0.9)	6.1 (0.9)	6.7 (1.1)	6.6 (0.9)	6.5 (0.9)	−0.04 (−0.24 to 0.16)	−0.04 (0.10)	0.686	−0.12 (−0.29 to 0.05)	−0.12 (0.09)	0.158
Nutritional status												
MNA	27.4 (2.1)	27.3 (2.2)	27.4 (2.0)	27.2 (2.2)	26.9 (2.1)	27.7 (1.9)	0.25 (−0.49 to 0.99)	0.25 (0.38)	0.511	−0.65 (−1.38 to 0.09)	−0.65 (0.38)	0.084
Psychological												
CES‐D	1.9 (3.1)	2.0 (3.8)	1.5 (4.0)	1.8 (3.9)	2.5 (5.4)	2.4 (4.8)	−0.64 (−2.07 to 0.80)	−0.64 (0.73)	0.384	−0.32 (−1.87 to 1.23)	−0.32 (0.79)	0.687
Social												
Social frailty	1.2 (1.2)	0.9 (0.9)	0.8 (1.2)	1.2 (1.3)	1.1 (1.2)	1.0 (1.2)	−0.16 (−0.68 to 0.36)	−0.16 (0.27)	0.549	0.00 (−0.49 to 0.49)	0.00 (0.25)	1.000
Personal mastery (Pearlin mastery score)	20.4 (2.9)	21.4 (4.2)	21.8 (3.3)	21.0 (3.3)	21.3 (3.3)	20.9 (3.2)	−0.68 (−2.07 to 0.70)	−0.68 (0.71)	0.335	−0.81 (−1.94 to 0.31)	−0.81 (0.57)	0.155
Cognition#												
MOCA												
Total score (score 0–30)	24.8 (4.2)	23.6 (5.6)	24.8 (4.8)	24.6 (3.5)	25.3 (3.5)	25.1 (4.5)	−1.84 (−3.11 to −0.57)	−1.84 (0.65)	0.005	1.32 (0.10–2.54)	1.32 (0.62)	0.034
Visuospatial/executive (score 0–5)	3.8 (1.1)	3.5 (1.5)	3.8 (1.4)	3.8 (1.2)	3.8 (1.1)	3.8 (1.3)	−0.25 (−0.76 to 0.26)	−0.25 (0.26)	0.339	0.30 (−0.14 to 0.73)	0.30 (0.22)	0.183
Naming (score 0–3)	2.6 (0.7)	2.6 (0.8)	2.6 (0.6)	2.6 (0.8)	2.8 (0.5)	2.8 (0.5)	−0.23 (−0.46 to 0.00)	−0.23 (0.12)	0.052	0.02 (−0.22 to 0.27)	0.02 (0.13)	0.857
Attention–the digit span (score 0–2)	1.9 (0.3)	1.8 (0.5)	1.8 (0.4)	1.8 (0.5)	1.8 (0.5)	1.8 (0.4)	−0.09 (−0.29 to 0.11)	−0.09 (0.10)	0.364	−0.02 (−0.24 to 0.19)	−0.02 (0.11)	0.834
Attention–number tapping test (score 0–1)	0.7 (0.5)	0.7 (0.5)	0.8 (0.4)	0.7 (0.5)	0.7 (0.5)	0.7 (0.5)	0.00 (−0.22 to 0.22)	0.00 (0.11)	1.000	0.05 (−0.16 to 0.25)	0.05 (0.11)	0.668
Attention–Serial 7 subtractions (score 0–3)	2.8 (0.5)	2.7 (0.6)	2.8 (0.4)	2.8 (0.5)	2.7 (0.7)	2.7 (0.6)	0.02 (−0.28 to 0.33)	0.02 (0.16)	0.885	0.07 (−0.23 to 0.36)	0.07 (0.15)	0.651
Language–sentence repetition (score 0–2)	1.6 (0.6)	1.3 (0.7)	1.3 (0.7)	1.6 (0.6)	1.5 (0.6)	1.3 (0.7)	−0.16 (−0.48 to 0.16)	−0.16 (0.16)	0.328	0.16 (−0.18 to 0.50)	0.16 (0.17)	0.359
Language–fluency (score 0–1)	0.8 (0.4)	0.7 (0.5)	0.9 (0.3)	0.8 (0.4)	0.9 (0.3)	0.8 (0.4)	−0.27 (−0.45 to −0.09)	−0.27 (0.09)	0.003	0.32 (0.13–0.51)	0.32 (0.10)	< 0.001
Abstraction (score 0–2)	1.3 (0.8)	1.3 (0.8)	1.3 (0.7)	1.3 (0.7)	1.5 (0.7)	1.5 (0.7)	−0.14 (−0.37 to 0.10)	−0.14 (0.12)	0.261	−0.09 (−0.33 to 0.14)	−0.09 (0.12)	0.448
Delayed recall (score 0–5)	2.9 (1.6)	2.9 (1.7)	3.1 (1.7)	2.8 (1.4)	3.3 (1.4)	3.3 (1.5)	−0.55 (−1.17 to 0.08)	−0.55 (0.32)	0.088	0.20 (−0.39 to 0.79)	0.20 (0.30)	0.497
Orientation (score 0–6)	5.8 (0.4)	5.6 (0.9)	5.8 (0.7)	5.9 (0.4)	5.8 (0.4)	5.8 (0.6)	−0.11 (−0.45 to 0.22)	−0.11 (0.17)	0.507	0.25 (−0.03 to 0.53)	0.25 (0.14)	0.080
Cardiometabolic												
Total cholesterol (mg/dL)	196.1 (33.1)	186.5 (34.8)	200.0 (44.0)	175.8 (34.8)	172.8 (31.7)	177.4 (36.5)	−6.51 (−16.10 to 4.00)	−6.51 (4.90)	0.185	8.86 (−3.36 to 21.08)	8.86 (6.24)	0.155
LDL‐C (mg/dL)	111.0 (28.8)	103.8 (23.9)	113.4 (35.1)	100.7 (29.7)	95.8 (25.0)	100.0 (30.9)	−2.33 (−10.26 to 5.61)	−2.33 (4.05)	0.566	5.47 (−4.84 to 15.77)	5.47 (5.26)	0.299
HDL‐C (mg/dL)	59.4 (16.0)	59.0 (14.3)	65.9 (18.7)	56.0 (15.6)	56.8 (16.2)	57.0 (16.3)	−1.12 (−4.25 to 2.02)	−1.12 (1.60)	0.485	6.65 (3.23 to 10.07)	6.65 (1.74)	<0.001
Triglyceride (mg/dL)	115.3 (89.5)	108.2 (50.6)	98.2 (43.7)	102.7 (47.4)	107.8 (60.9)	113.9 (88.3)	−12.19 (−34.10 to 9.73)	−12.19 (11.18)	0.276	−16.07 (−30.01 to −2.04)	−16.07 (7.16)	0.025
Glucose (mg/dL)	100.0 (16.2)	101.0 (18.9)	104.1 (19.2)	109.5 (33.3)	109.0 (42.5)	108.5 (29.3)	1.49 (−6.75 to 9.72)	1.49 (4.20)	0.723	3.56 (−7.30 to 14.42)	3.56 (5.54)	0.521
HbA1c	5.9 (0.6)	5.9 (0.6)	6.0 (0.6)	6.3 (1.1)	6.2 (1.1)	6.2 (0.9)	0.05 (−0.05 to 0.15)	0.05 (0.05)	0.300	0.10 (−0.07 to 0.27)	0.10 (0.09)	0.232

*Note:* All models adjusted for site, age and BMI; except for cognitive performance: # adjusted for site, age, education and BMI.

Abbreviations: CES‐D, the Center for Epidemiologic Studies Depression; HDL‐C, high‐density lipoprotein cholesterol; HgbA1c, hemoglobinA1c; LDL‐C, low‐density lipoprotein cholesterol; RASM, relative appendicular skeletal muscle mass.

Cognitive measures also showed significant changes:the intervention group had lower MoCA total scores (−1.84 points, *p* = 0.005) at 6th month, but these scores improved significantly at the 12th month (MoCA: +1.32 points, *p* = 0.034), after adjustment for site, age, BMI and education level. Among the cognitive domains, verbal fluency (language domain) exhibited the most significant changes between groups at both 6th and 12th months. A significant decrease in verbal fluency scores was observed in the intervention group at the 6‐month assessment compared with the control group (−0.27 points, *p* = 0.003), which subsequently reversed to a significant improvement of +0.32 points at the 12‐month follow‐up (*p* < 0.001).

Despite improved muscular function, as evidenced by improved 5‐time chair rise tests at the 6‐month assessment, the RASM did not show significant changes between groups. Nevertheless, a significant increase in total body fat (+1.77 kg, *p* = 0.041) was observed in the intervention group compared with the control group at the 12‐month follow‐up. Cardiometabolic outcomes showed improvement in the intervention group, with increased HDL‐C (+6.65 mg/dL, *p* < 0.001) and decreased triglycerides (−16.07 mg/dL, *p* = 0.025) at 12th month compared with that at the 6th month.

This study also examined intervention effects in sub‐cohorts: Yi‐Lan (rural, *n* = 39) (Table 3) and Taipei (urban, *n* = 49) (Table 4). A significant decline in MoCA scores was observed in the Yi‐Lan sub‐cohort (Table 3) intervention group at the 6‐month assessment (−3.09 points, *p* = 0.001), which subsequently reversed to a significant improvement of +3.06 points at the 12‐month follow‐up (*p* = 0.007). Body composition alterations at 12 months included reductions in body muscle mass (−1.32 kg, *p* < 0.001) and skeletal muscle mass (−0.25 kg, *p* = 0.009), concurrent with increases in body fat mass (+2.39 kg, *p* = 0.022) and total body fat percentage (+3.77%, *p* = 0.004), while HDL‐C levels demonstrated a significant increase (+9.51 mg/dL, *p* < 0.001).

**TABLE 3:** Intervention effect on physical, psychosocial, cognitive and cardiometabolic outcomes in Yi‐Lan cohort.

Outcome	Intervention (*n* = 21)			Control (*n* = 18)			Group comparison at 6 M (vs. baseline)			Group comparison at 12 M (vs. 6 M)		
	Baseline	6 M	12 M	Baseline	6 M	12 M	Difference in group means (95% CI)	Beta (SE)	*p*‐value	Difference in group means (95% CI)	Beta (SE)	*p*‐value
Physical performance												
5‐time chair rise test (s)	10.7 (2.8)	10.0 (3.6)	9 (2.3)	12.2 (4.5)	11.8 (4.8)	10.3 (3.5)	−0.30 (−2.19 to 1.60)	−0.30 (0.97)	NS	0.44 (−1.19 to −2.09)	0.44 (0.84)	NS
6‐m walk (s)	1.0 (0.2)	1.0 (0.2)	1.1 (0.2)	0.9 (0.2)	1.0 (0.2)	1.0 (0.2)	−0.12 (−0.28 to 0.05)	−0.12 (0.08)	NS	0.11 (−0.02 to 0.23)	0.11 (0.06)	NS
Hand grip strength (kg)	21.4 (6.7)	21.3 (7.8)	21.8 (7.3)	22.4 (6.6)	24.0 (5.7)	23.3 (7.3)	−1.71 (−4.22 to 0.80)	−1.71 (1.28)	NS	1.23 (−0.41 to 2.88)	1.23 (0.84)	NS
Frailty (CFS)	2.7 (0.7)	2.6 (0.8)	2.7 (0.7)	2.7 (0.8)	2.9 (0.3)	2.7 (0.7)	−0.32 (−0.65 to 0.02)	−0.32 (0.17)	NS	0.31 (0.00 to 0.61)	0.31 (0.16)	NS
Body composition												
Body muscle mass (kg)	21.1 (4.8)	20.2 (4.3)	19.7 (4.4)	22.4 (5.1)	20.8 (3.9)	21.6 (3.8)	0.70 (−0.57 to 1.96)	0.70 (0.64)	NS	−1.32 (−2.10 to −0.54)	−1.32 (0.40)	< 0.001
Body fat mass (kg)	19.0 (5.9)	18.8 (5.5)	20.1 (5.0)	21.9 (7.9)	24.1 (8.1)	23.0 (7.4)	−2.39 (−5.27 to 0.49)	−2.39 (1.47)	NS	2.39 (0.35 to 4.44)	2.39 (1.04)	0.022
Skeletal muscle mass (kg)	2.4 (0.4)	2.4 (0.4)	2.3 (0.4)	3.7 (4.9)	2.2 (0.5)	2.4 (0.4)	1.40 (−0.77 to 3.58)	1.40 (1.11)	NS	−0.25 (−0.44 to 0.06)	−0.25 (0.10)	0.009
Total body fat (%)	32.3 (6.8)	32.7 (5.4)	35.0 (5.1)	34.0 (9.0)	37.2 (7.3)	35.8 (6.9)	−2.76 (−6.64 to 0.82)	−2.76 (0.82)	NS	3.77 (1.21 to 6.33)	3.77 (1.31)	0.004
RASM (kg/m^2^)	6.8 (1.2)	6.3 (1.1)	6.0 (1.1)	6.9 (1.2)	6.6 (0.8)	6.5 (0.9)	−0.16 (−0.49 to 0.18)	−0.15 (0.17)	NS	−0.18 (−0.37 to 0.00)	−0.18 (0.09)	NS
Nutritional status												
MNA	27.0 (2.5)	26.7 (2.6)	27.4 (2.3)	27.1 (2.2)	26.7 (2.4)	27.8 (1.8)	0.00 (−1.24 to 1.25)	0.00 (0.63)	NS	−0.39 (−1.42 to 0.64)	−0.39 (0.53)	NS
Psychological												
CES‐D	2.5 (3.9)	2.0 (3.6)	2.4 (5.2)	1.1 (1.3)	1.6 (3.8)	3.0 (6.3)	−1.07 (−3.42 to 1.28)	−1.07 (1.20)	NS	−1.02 (−3.29 to 1.26)	−1.02 (1.16)	NS
Social												
Social frailty	1.9 (1.2)	1.0 (1.1)	1.1 (1.3)	1.8 (1.3)	1.1 (1.3)	1.4 (1.5)	−0.18 (−0.96 to 0.60)	−0.18 (0.39)	NS	−0.19 (−0.88 to 0.50)	−0.19 (0.35)	NS
Personal mastery (Pearlin mastery score)	22.5 (4.0)	23.1 (4.0)	22.3 (3.8)	21.9 (3.3)	23.9 (4.5)	24.2 (2.5)	−1.44 (−4.12 to 1.25)	−1.44 (1.37)	NS	−1.13 (−3.21 to 0.95)	−1.13 (1.06)	NS
Cognition#												
MOCA												
Total score (score 0–30)	23.5 (5.0)	20.4 (6.1)	22.7 (5.2)	22.9 (4.3)	22.8 (3.9)	22.1 (5.4)	−3.09 (−4.98 to −1.19)	−3.09 (0.97)	0.001	3.06 (0.84–5.27)	3.06 (1.13)	0.007
Cardiometabolic												
Total cholesterol (mg/dL)	193.8 (36.5)	179.2 (35.9)	205.3 (50.2)	173.4 (32.1)	169.5 (35.3)	177.0 (35.6)	−10.67 (−28.14 to 6.81)	−10.67 (8.91)	NS	18.58 (−1.91 to 39.07)	18.58 (10.45)	NS
LDL‐C (mg/dL)	100.0 (29.4)	91.1 (27.4)	109.0 (39.0)	102.9 (29.5)	95.0 (24.7)	101.2 (25.1)	−0.96 (−15.24 to 13.32)	−0.96 (7.29)	NS	11.66 (−5.45 to 28.78)	11.66 (8.73)	NS
HDL‐C (mg/dL)	61.3 (17.5)	60.6 (15.4)	71.1 (20.1)	52.9 (11.6)	55.3 (14.6)	56.2 (15.5)	−3.06 (−7.79 to 1.66)	−3.06 (2.41)	NS	9.51 (3.88 to 15. 14)	9.51 (2.87)	< 0.001
Triglyceride (mg/dL)	137.8 (121.4)	115.4 (64.6)	107.2 (51.7)	104.2 (41.8)	102.7 (35.0)	110.0 (46.7)	−20.92 (−60.51 to 18.67)	−20.92 (20.20)	NS	−15.02 (−33.12 to 3.07)	−15.02 (9.23)	NS
Glucose (mg/dL)	98.2 (14.0)	103.4 (22.6)	107.0 (21.9)	115.6 (41.6)	119.4 (61.3)	115.3 (36.3)	1.33 (−12.91 to 15.57)	1.33 (7.27)	NS	7.72 (−16.86 to 32.30)	7.72 (12.54)	NS
HbA1c	5.9 (0.6)	5.9 (0.6)	6.0 (0.6)	6.5 (1.6)	6.5 (1.5)	6.4 (1.1)	0.03 (−0.10 to 0.17)	0.03 (0.07)	NS	0.19 (−0.19 to 0.56)	0.19 (0.19)	NS

*Note:* All models adjusted for age and BMI; except for cognitive performance: # adjusted for age, education and BMI.

Abbreviations: CES‐D, the Center for Epidemiologic Studies Depression; HDL‐C, high‐density lipoprotein cholesterol; HgbA1c, hemoglobinA1c; LDL‐C, low‐density lipoprotein cholesterol; NS, non‐statistical significant; RASM, relative appendicular skeletal muscle mass.

**TABLE 4:** Intervention effect on physical, psychosocial, cognitive and cardiometabolic outcomes in Taipei cohort.

Outcome	Intervention (*n* = 23)			Control (*n* = 26)			Group comparison at 6 M (vs. baseline)			Group comparison at 12 M (vs. 6 M)		
	Baseline	6 M	12 M	Baseline	6 M	12 M	Difference in group means (95% CI)	Beta (SE)	*p*‐value	Difference in group means (95% CI)	Beta (SE)	*p*‐value
Physical performance												
5‐time chair rise test (s)	9.5 (2.7)	8.4 (1.3)	8.4 (2.0)	8.2 (1.4)	8.9 (2.2)	8.9 (2.9)	−1.85 (−3.07 to −0.64)	−1.85 (0.62)	0.003	0.03 (−0.97 to 1.03)	0.03 (0.51)	NS
6‐m walk (s)	1.2 (0.2)	1.3 (0.2)	1.3 (0.1)	1.3 (0.3)	1.3 (0.2)	1.3 (0.2)	0.12 (−0.02 to 0.25)	0.12 (0.07)	NS	−0.05 (−0.16 to 0.06)	−0.05 (0.05)	NS
Hand grip strength (kg)	22.4 (7.8)	23.8 (7.4)	23.7 (7.6)	22.4 (6.8)	22.2 (7.6)	23.4 (6.7)	1.57 (−0.14 to 3.27)	1.57 (0.87)	NS	−1.27 (−2.71 to 0.18)	−1.27 (0.74)	NS
Frailty (CFS)	2.3 (0.5)	2.2 (0.4)	2.3 (0.5)	2.3 (0.7)	2.3 (0.6)	2.2 (0.7)	−0.05 (−0.22 to 0.13)	−0.05 (0.09)	NS	0.24 (0.06–0.42)	0.24 (0.09)	0.009
Body composition												
Body muscle mass (kg)	21.3 (4.1)	21.9 (3.8)	21.0 (3.7)	22.2 (4.5)	22.3 (4.5)	22.1 (4.2)	0.46 (0.09–0.84)	0.46 (0.19)	0.016	−0.66 (−1.11 to −0.22)	−0.66 (0.23)	0.004
Body fat mass (kg)	18.6 (3.9)	17.2 (4.3)	18.7 (4.0)	20.4 (6.4)	19.5 (6.1)	20.2 (6.6)	−0.50 (−1.30 to 0.30)	−0.50 (0.41)	NS	0.87 (−0.08 to 1.81)	0.87 (0.48)	NS
Skeletal muscle mass (kg)	2.4 (0.4)	2.4 (0.3)	2.4 (0.3)	2.4 (0.4)	2.4 (0.4)	2.4 (0.4)	0.02 (−0.03 to 0.08)	0.02 (0.03)	NS	−0.05 (−0.11 to 0.02)	−0.05 (0.03)	NS
Total body fat (%)	32.0 (5.7)	29.6 (5.5)	32.2 (5.2)	33.0 (7.8)	30.7 (8.8)	32.8 (8.0)	−0.08 (−1.93 to 1.76)	−0.08 (0.94)	NS	0.41 (−1.66 to 2.47)	0.41 (1.05)	NS
RASM (kg/m^2^)	6.1 (0.8)	6.2 (0.7)	6.1 (0.8)	6.6 (1.0)	6.6 (1.0)	6.5 (1.0)	0.11 (−0.03 to 0.25)	0.11 (0.07)	NS	−0.07 (−0.35 to 0.20)	−0.07 (0.14)	NS
Nutritional status												
MNA	27.7 (1.6)	27.9 (1.6)	27.5 (1.6)	27.3 (2.3)	27.0 (1.9)	27.6 (2.0)	0.48 (−0.40 to 1.36)	0.48 (0.45)	NS	−0.95 (−1.94 to 0.04)	−0.95 (0.50)	NS
Psychological												
CES‐D	1.3 (2.2)	2.0 (4.1)	0.7 (2.3)	2.3 (5.0)	3.2 (6.3)	2.0 (3.5)	−0.18 (−1.88 to 1.50)	−0.18 (0.86)	NS	−0.04 (−1.86 to 1.79)	−0.04 (0.93)	NS
Social												
Social frailty	0.5 (0.7)	0.9 (0.7)	0.6 (1.1)	0.8 (1.2)	1.2 (1.1)	0.7 (0.9)	0.01 (−0.52 to 0.54)	0.01 (0.27)	NS	0.08 (−0.56 to 0.71)	0.08 (0.32)	NS
Personal mastery (Pearlin mastery score)	19.5 (1.6)	19.5 (2.3)	19.6 (1.7)	19.3 (2.1)	19.6 (2.7)	20.1 (2.7)	−0.23 (−1.48 to 1.02)	−0.23 (0.64)	NS	−0.50 (−1.61 to 0.62)	−0.50 (0.57)	NS
Cognition#												
MOCA												
Total score (score 0–30)	25.9 (2.9)	26.5 (3.1)	26.7 (3.5)	25.8 (2.2)	26.9 (1.9)	27.2 (1.8)	−0.55 (−1.86 to 0.78)	−0.55 (0.68)	NS	−0.13 (−1.24 to 0.98)	−0.13 (0.57)	NS
Cardiometabolic												
Total cholesterol (mg/dL)	198.1 (31.7)	192.9 (33.4)	195.4 (38.4)	177.4 (37.0)	174.9 (29.6)	177.6 (37.7)	−2.72 (−13.14 to 7.70)	−2.72 (5.31)	NS	−0.21 (−12.55 to 12.14)	−0.21 (6.30)	NS
LDL‐C (mg/dL)	120.5 (25.2)	114.9 (30.0)	117.2 (31.9)	99.2 (30.3)	96.4 (25.7)	99.1 (34.6)	−2.84 (−11.82 to 6.15)	−2.84 (4.59)	NS	−0.38 (−11.62 to 10.86)	−0.38 (5.73)	NS
HDL‐C (mg/dL)	57.8 (14.7)	57.7 (13.4)	61.5 (16.4)	58.1 (17.7)	57.8 (17.4)	57.6 (17.1)	0.22 (−3.77 to 4.20)	0.22 (2.03)	NS	4.02 (0.21 to 7.82)	4.02 (1.94)	NS
Triglyceride (mg/dL)	95.7 (41.2)	102.0 (34.5)	90.4 (34.8)	101.7 (51.5)	111.2 (73.6)	116.7 (108.1)	−3.15 (−22.89 to 16.60)	−3.15 (10.07)	NS	−17.04 (−36.94 to 2.64)	−17.04 (10.10)	NS
Glucose (mg/dL)	101.7 (18.1)	99.0 (15.3)	101.7 (16.6)	105.5 (26.6)	102.2 (22.5)	104.1 (23.4)	0.70 (−8.61 to 10.00)	0.70 (4.75)	NS	0.72 (−6.04 to 7.49)	0.72 (3.45)	NS
HbA1c	5.9 (0.6)	5.9 (0.7)	5.9 (0.7)	6.1 (0.6)	6.0 (0.7)	6.1 (0.7)	0.06 (−0.07 to 0.19)	0.06 (0.07)	NS	0.05 (−0.07 to 0.17)	0.06 (0.06)	NS

*Note:* All models adjusted for age and BMI; except for cognitive performance: # adjusted for age, education and BMI.

Abbreviations: CES‐D, the Center for Epidemiologic Studies Depression; HDL‐C, high‐density lipoprotein cholesterol; HgbA1c, hemoglobinA1c; LDL‐C, low‐density lipoprotein cholesterol; NS, non‐statistical significant; RASM, relative appendicular skeletal muscle mass.

In contrast, the Taipei (urban) sub‐cohort showed different patterns (Table 4). Physical performance improved significantly, with the time of 5‐time chair rise test decreasing by 1.85 s (*p* = 0.003) at 6th month in the intervention group.

## 4 Discussion

In the ENHANCE trial of twice‐weekly multidomain sessions combining physical exercise, cognitive training and nutrition education, MRI analysis revealed that the intervention group demonstrated significantly less GMV reduction in the left inferior temporal lobe compared with controls over 12 months, addressing a critical gap in previous literature that often lacked neuroimaging evidence. Further rural and urban analysis uncovered distinct geographical effects: rural participants showed preserved brain volume in the cerebellum and occipital cortex with greater cognitive improvements, while urban participants demonstrated superior physical performance gains despite greater volume reduction in the left temporal‐occipital fusiform cortex. These regional differences in structural and functional outcomes suggest that sociogeographical factors significantly influence intervention effectiveness, highlighting the importance of tailored implementation strategies.

Our findings build upon and complement the seminal FINGER trial, which established the efficacy of multidomain interventions in preventing cognitive decline [5]. In contrast to FINGER's exploratory brain MRI sub‐study, which was limited by scanner heterogeneity and reduced sensitivity of predefined ROI measurements, our voxel‐wise whole‐brain analysis demonstrated intervention‐specific neuroplasticity that correlated with cognitive outcomes. On the other hand, while FINGER primarily examined hippocampal volume and beta‐amyloid deposition, revealing cognitive improvements predominantly in non‐memory domains [26], our study identified broader structural changes that provide anatomical substrates for the observed clinical improvements. Notably, we extended FINGER's findings by establishing associations between physical performance measures, particularly the 5‐time chair‐rise test, and structural brain changes, suggesting its potential utility as a neurobiological marker for intervention efficacy. Furthermore, our analysis of urban–rural disparities in intervention outcomes addresses a critical gap in implementation research, providing essential insights for adapting FINGER‐based multidomain interventions across diverse populations and healthcare settings.

In previous RCTs involving multidomain interventions, improvements have been observed in global cognitive performance and specific cognitive domains, such as visuospatial executive functions [4, 8, 10]. Regarding physical functions, these trials have shown improvements in frailty scores among older adults, although no significant gains in gait speed or handgrip strength were noted [4, 8, 10]. The ENHANCE trial, which increased intervention frequency to twice weekly over 12 months, demonstrated significant improvements in both physical and cognitive performance, as evidenced by the 5‐time chair rise test and global neuropsychological assessments. Noteworthy, we found that the THISCE trial identified substantial benefits primarily in the older subgroup, while both the THISCE and TIGER trials reported a decline in physical performance gains over time, even as cognitive improvements were delayed but sustained [4, 10]. In the ENHANCE trial, physical performance gains (chair‐rise tests) were maintained, though cognitive improvements continued to be delayed as the previous trials. Besides, this trial is the first, employing MRI‐detected brain structural changes as the primary outcome to discover the neuroplasticity effects from a multidomain intervention. While a few of RCTs focusing on physical or nutrition‐related interventions have reported promising effects on brain GMV measures [27, 28], the impact of multidomain interventions on structural brain changes remains unclear.

While weakness and slowness are established risk factors for cognitive impairment and dementia in middle and late adulthood [13, 14], physical exercise confers benefits on cognitive function and brain health among them [29]. Findings from these epidemiological and interventional studies corroborate the hypothesized age‐related muscle‐to‐brain axis [30], indicating potential interactions between muscle aging and brain function in older populations [31]. The current trial demonstrated that a multidomain intervention improved physical function and cognitive abilities, specifically verbal fluency, in at‐risk older adults. Furthermore, observed alterations in brain structure align with these cognitive enhancements, supporting the hypothesized muscle‐to‐brain axis and suggesting a potential link between muscle and brain health. The left inferior temporal lobe, a brain region previously associated with diverse cognitive functions including language, semantic memory, visual perception and multisensory integration [32, 33, 34], appears to be integral to critical physical and cognitive aspects of aging. Our findings suggest that this region is positively influenced by multidomain intervention, potentially via a muscle‐to‐brain axis. Subgroup analyses demonstrated marked heterogeneity in neuroplastic responses to multidomain interventions based on urban–rural residence. Cognitive enhancements were more prevalent in rural participants, whereas physical improvements were more pronounced in urban counterparts. These findings highlight the complexity of promoting healthy aging across diverse populations, suggesting that a standardized multidomain intervention may not optimally address the specific needs of different sociocultural environments. Rural participants, characterized by older age and lower education, exhibited greater cognitive gains from the intervention compared with younger, more educated urban counterparts, potentially due to lower baseline cognitive function and higher physical activity levels in rural residents, while urban participants demonstrated more pronounced physical improvements. These disparities were evident in the neuroimaging results as well. Rural participants exhibited significantly preserved GMV across brain regions, which correlated with their cognitive improvements. In contrast, urban participants showed greater GMV reduction in the left temporal‐occipital fusiform cortex, corresponding to the lack of enhanced intervention effects on their cognitive functions. The urban–rural disparities observed in neuroplastic responses to multidomain interventions suggest that socio‐environmental factors significantly influence brain plasticity. While the multidomain intervention employed a comprehensive approach to enhance both physical and cognitive health, the observed disparities in neuroplasticity across urban and rural settings underscore the influence of sociocultural heterogeneity. To maximize intervention efficacy, a more nuanced approach is required, tailoring intervention strategies to the specific needs and characteristics of different community contexts.

Notwithstanding extensive research efforts, several limitations persist in the present study. First, the COVID‐19 pandemic imposed challenges on recruitment of study participant and their adherence to the follow‐up brain MRI examinations. Furthermore, the pandemic may have impact on participants' restricted daily activities, which may have contributed to adverse shifts in body composition and functional ability; however, the intervention demonstrated efficacy in not only preserving but also enhancing physical performance. Second, this study represents the first trial of multidomain intervention using neuroimaging changes as the primary outcome. In contrast to previous studies that employed region‐of‐interest approaches targeting predetermined brain areas, our implementation of advanced voxel‐wise analysis enabled detection of subtle volumetric changes throughout the entire brain. While this methodological advancement enhances sensitivity to intervention‐induced neuroplasticity, it introduces challenges in comparative analysis with existing literature and precludes the use of neuroimaging metrics for a priori sample size estimation. Hence, we calculated the sample size using secondary clinical measures, which are more directly linked to the expected intervention effects. However, subsequent investigations with expanded sample sizes and stratified urban–rural cohorts are necessary to further elucidate the findings of this study. Third, neuropsychological tests are known to exhibit learning effects across all cognitive intervention trials. In our study, bidirectional changes in MoCA scores and improvements in the control group likely reflect learning effects, a well‐documented phenomenon in cognitive testing. This observation reinforces our strategic decision to utilize neuroimaging as the primary outcome measure, which provides more objective evidence of intervention‐induced brain changes that are not subject to practice effects. Lastly, while participants with established dementia or significantly impaired neuropsychological function were excluded, the absence of comprehensive preclinical cognitive assessments limits our ability to definitively categorize participants. Consequently, the study population may encompass individuals with mild cognitive impairment or early‐stage dementia, potentially obscuring differential treatment effects across distinct preclinical phenotypes. However, it is almost universal in the trials targeted at community‐dwelling older adults, especially including those from rural communities.

In conclusion, this pioneering trial investigated the impact of a 12‐month multidomain intervention on physical and cognitive function, accompanied by brain structural alterations assessed via MRI, to elucidate the neurobiological mechanisms underlying healthy aging. The marked disparities in response between urban and rural participants underscore the profound influence of socioenvironmental factors on intervention outcomes. These findings emphasize the necessity for tailored interventions to optimize healthy aging across diverse populations, laying the groundwork for the development of targeted strategies to enhance both physical and cognitive well‐being in later life.

## Conflicts of Interest

The authors declare no conflicts of interest.

## Supporting information


**Table S1.** Demographics of study participants of MRI analysis.
**Table S2.** Anatomical region with significant altered volume change rate in intervention group and control group.
