# Supplementary material for: Enhancing Neurocognitive Health via Activity, Nutrition and Cognitive Exercise (ENHANCE): A Randomized Controlled Trial
Source: J Cachexia Sarcopenia Muscle. 2025 Jun 4;16(3):e13830. doi: 10.1002/jcsm.13830 (PMC12134766; doi:10.1002/jcsm.13830)
Supplement: Supplementary file 1 — Table S1. Demographics of study participants of MRI analysis. Table S2. Anatomical region with significant altered volume change rate in intervention group and control group. [file JCSM-16-e13830-s001.docx]

**Supplementary Data**

**Supplemental methods: brain MRI acquisition and analytic methods**

1. Validation of Tensor-Based Morphometry (TBM) Techniques:
The TBM methodology applied in our study has been extensively validated in prior research. TBM is an image analysis technique that measures brain structural differences from the gradients of deformation fields that align one image to another [1-3]. Furthermore, Ashburner and Ridgway (2013) specifically highlighted the superiority of TBM for detecting voxel-wise changes in gray matter volume (GMV) over time [4]. This methodology has been extensively applied to study brain plasticity resulting from training interventions [5] and to monitor brain atrophy during disease progression [6,7]. These studies affirm the methodological rigor and sensitivity of TBM in capturing neuroplasticity effects, even in populations with subtle or heterogeneous structural changes, such as older adults.

2. Quality Assurance in Neuroimage data:
To ensure the reliability of our neuroimaging data, all MRI images underwent visual evaluation by an experienced neuroradiologist to identify and exclude images with artifacts, motion distortions, or other anomalies that could compromise data integrity. Additionally, each preprocessing step was manually inspected to verify accuracy and consistency. This rigorous approach ensured the robustness and validity of our findings.

3. Reliability of Analytical Approaches:
The voxel-wise analysis approach employed in our study was rigorously designed to minimize false positives and maximize the sensitivity of detecting group differences. We applied family-wise error (FWE) correction to control for multiple comparisons, ensuring that the observed results were statistically robust.

4. Reproducibility and Transparency:
Recognizing the importance of reproducibility, we have made all unthresholded voxel-wise statistical maps publicly available via the NeuroVault platform (<https://neurovault.org/collections/16106/>). This enables external researchers to validate and build upon our findings, thereby enhancing the scientific integrity of our results.

In conclusion, the advanced MRI techniques employed in this study are grounded in validated frameworks, rigorously applied quality control measures, and alignment with best practices in neuroimaging. Additionally, our team includes four current co-authors (Dr. Ching-Po Lin, Dr. Kun-Hsien Chou and Dr. Pei-Lin Lee from the Brain Connectivity Lab in National Yang Ming Chiao Tung University and Dr. Chih-Ping Chung from the Neurological Institute of Taipei Veterans General Hospital) who have long-standing expertise in neuroimaging research, further strengthening the methodological foundation of our study. We believe this ensures the accuracy and reliability of the reported findings.

[1] Freeborough, Peter A., and Nick C. Fox. "Modeling brain deformations in Alzheimer disease by fluid registration of serial 3D MR images." Journal of computer assisted tomography 22.5 (1998): 838-843.

[2] Riddle, W.R., Li, R., Fitzpatrick, J.M., DonLevy, S.C., Dawant, B.M., Price, R.R., 2004. Characterizing changes in MR images with color-coded Jacobians. Magn. Reson. Imaging 22, 769–777.
[3] Thompson, P.M., Giedd, J.N., Woods, R.P., MacDonald, D., Evans, A.C., Toga, A.W., 2000. Growth patterns in the developing brain detected by using continuum mechanical tensor maps. Nature 404, 190–193.
[4] Ashburner, John, and Gerard R. Ridgway. "Symmetric diffeomorphic modeling of longitudinal structural MRI." Frontiers in neuroscience 6 (2013): 197.
[5] Colom, Roberto, et al. "Brain structural changes following adaptive cognitive training assessed by Tensor-Based Morphometry (TBM)." Neuropsychologia 91 (2016): 77-85.
[6] Brambati, S. M., et al. "Atrophy progression in semantic dementia with asymmetric temporal involvement: a tensor-based morphometry study." Neurobiology of aging 30.1 (2009): 103-111.
[7] Hua, Xue, et al. "Unbiased tensor-based morphometry: improved robustness and sample size estimates for Alzheimer's disease clinical trials." Neuroimage 66 (2013): 648-661.

**Supplementary Table 1 Demographics of study participants of MRI analysis**

|  |  | **Total population** | | | |  | **Yi-Lan cohort** | | | |  | **Taipei cohort** | | | |
| --- | --- | --- | --- | --- | --- | --- | --- | --- | --- | --- | --- | --- | --- | --- | --- |
|  |  | Total  (n=76) | Intervention  (n=35) | Control  (n=41) | p-value |  | Total  (n=27) | Intervention  (n=12) | Control  (n=15) | p-value |  | Total  (n=49) | Intervention  (n=23) | Control  (n=26) | p-value |
| Site, n (%) | |  |  |  | 0.631 |  |  |  |  |  |  |  |  |  |  |
|  | *Yi-Lan* | 26 (35.6%) | 11 (32.4%) | 15 (38.5%) |  |  |  |  |  |  |  |  |  |  |  |
|  | *Taipei* | 47 (64.4%) | 23 (67.7%) | 24 (64.5%) |  |  |  |  |  |  |  |  |  |  |  |
| Age (years), mean (SD) | | 73.3 (4.9) | 75.0 (5.3) | 71.9 (4.1) | 0.005 |  | 73.1 (5.5) | 74.6 (6.3) | 72.0 (4.8) | 0.249 |  | 73.4 (4.6) | 75.2 (4.9) | 71.7 (3.7) | 0.008 |
| Sex, n (%) | |  |  |  | 0.791 |  |  |  |  | 0.053 |  |  |  |  | 0.534 |
|  | *Male* | 19 (26.0%) | 8 (23.5%) | 13 (29.6%) |  |  | 5 (19.2%) | 0 (0%) | 5 (33.3%) |  |  | 14 (29.8%) | 8 (34.8%) | 6 (25.0%) |  |
|  | *Female* | 54 (74.0%) | 26 (76.5%) | 31 (70.5%) |  |  | 21 (80.8%) | 11 (100%) | 10 (66.7%) |  |  | 33 (70.2%) | 15 (65.2%) | 18 (75.0%) |  |
| Education year, n (%) | |  |  |  | 0.791 |  |  |  |  | 0.701 |  |  |  |  | 0.609 |
|  | *< 9* | 19 (26.0%) | 8 (23.5%) | 11 (28.2%) |  |  | 15 (57.7%) | 7 (63.6%) | 8 (53.3%) |  |  | 4 (8.5%) | 1 (4.3%) | 3 (12.5%) |  |
|  | *≥ 9* | 54 (74.0%) | 26 (76.5%) | 28 (71.8%) |  |  | 11 (42.3%) | 4 (36.4%) | 7 (46.7%) |  |  | 43 (91.5%) | 22 (95.7%) | 21 (87.5%) |  |
| Anthropometric Measurements | |  |  |  |  |  |  |  |  |  |  |  |  |  |  |
|  | *BMI (kg/m^2^), mean (SD)* | 24.4 (3.5) | 23.2 (2.6) | 25.4 (3.9) | 0.008 |  | 25.0 (3.9) | 23.7 (3.1) | 25.9 (4.2) | 0.161 |  | 24.0 (3.2) | 23.0 (2.3) | 25.0 (3.7) | 0.031 |
| MRI measurements (L), mean (SD) | |  |  |  |  |  |  |  |  |  |  |  |  |  |  |
|  | *Total intracranial volume* | 1.375 (0.115) | 1.367 (0.132) | 1.383 (0.098) | 0.561 |  | 1.343 (0.110) | 1.314 (0.091) | 1.364 (0.121) | 0.262 |  | 1.393 (0.114) | 1.392 (0.143) | 1.395 (0.082) | 0.948 |
|  | *Gray matter volume* | 0.599 (0.050) | 0.591 (0.047) | 0.606 (0.052) | 0.198 |  | 0.598 (0.062) | 0.587 (0.049) | 0.606 (0.070) | 0.451 |  | 0.600 (0.043) | 0.593 (0.046) | 0.606 (0.039) | 0.287 |
|  | *White matter volume* | 0.399 (0.046) | 0.395 (0.050) | 0.402 (0.043) | 0.516 |  | 0.387 (0.048) | 0.379 (0.041) | 0.392 (0.053) | 0.501 |  | 0.405 (0.044) | 0.402 (0.053) | 0.408 (0.034) | 0.667 |
|  | *Cerebrospinal fluid volume* | 0.378 (0.071) | 0.381 (0.080) | 0.375 (0.064) | 0.702 |  | 0.359 (0.069) | 0.349 (0.059) | 0.366 (0.077) | 0.530 |  | 0.388 (0.071) | 0.397 (0.085) | 0.380 (0.057) | 0.428 |

Abbreviations: BMI, body mass index; CCI, Charlson comorbidity index, COPD, chronic obstructive pulmonary disease.

**Supplementary Table 2. Anatomical region with significant altered volume change rate in intervention group and control group.**

| **MNI coordinate (x, y, z)** | **Cluster size (mm^3^)** | **Maximum t-value** | **p-value** | **Anatomical region** |  | **Volume change rate (a.u.) [mean ± SD]** | | | | | | | |
| --- | --- | --- | --- | --- | --- | --- | --- | --- | --- | --- | --- | --- | --- |
|  |  |  |  |  |  | Total population | |  | Yi-Lan cohort | |  | Taipei cohort | |
|  |  |  |  |  |  | *Intervention* | *Control* |  | *Intervention* | *Control* |  | *Intervention* | *Control* |
| *Total population: Intervention group > Control group* | | | | |  |  |  |  |  |  |  |  |  |
| -44, -21, -29 | 986 | 3.45 | <0.001 | Left inferior temporal gyrus |  | 0.0015±0.0067 | -0.044±0.0075 |  | 0.0031±0.0074 | -0.0062±0.0067 |  | 0.008±0.0064 | -0.0033±0.0079 |
| *Yi-Lan cohort: Intervention group > Control group* | | | | |  |  |  |  |  |  |  |  |  |
| -26, -90, 23 | 2531 | 4.23 | <0.001 | Left occipital pole |  | 0.0034±0.0084 | 0.0019±0.0125 |  | 0.0078±0.0068 | -0.0049±0.0100 |  | 0.0013±0.0084 | 0.0062±0.0120 |
| 45, -77, -30 | 1154 | 4.13 | <0.001 | Right cerebellum crus I |  | 0.0058±0.0088 | 0.0012±0.0092 |  | 0.0114±0.0098 | -0.0008±0.0092 |  | 0.0031±0.0070 | 0.0025±0.0092 |
| -59, -48, -24 | 1704 | 4.07 | <0.001 | Left inferior temporal gyrus |  | 0.0026±0.0065 | -0.0011±0.0082 |  | 0.0071±0.0026 | -0.0044±0.0075 |  | 0.0005±0.0068 | 0.0010±0.0081 |
| 18, -89, -32 | 1863 | 3.94 | <0.001 | Right cerebellum crus II |  | 0.0047±0.0107 | 0.0013±0.0085 |  | 0.0136±0.0071 | -0.0012±0.0105 |  | 0.0004±0.0095 | 0.0028±0.0069 |
| -48, -69, -29 | 1505 | 3.76 | <0.001 | Left cerebellum crus I |  | 0.0008±0.0086 | -0.0017±0.0106 |  | 0.0061±0.0054 | -0.0053±0.0124 |  | -0.0017±0.0088 | 0.0005±0.0089 |
| 53, -69, 1 | 1461 | 3.64 | <0.001 | Right inferior lateral occipital cortex |  | 0.0021±0.0098 | 0.0004±0.0101 |  | 0.0081±0.0081 | -0.0048±0.0087 |  | -0.0008±0.0094 | 0.0037±0.0096 |
| 31, -71, 41 | 844 | 3.27 | 0.001 | Right superior lateral occipital cortex |  | 0.0018±0.0100 | 0.0008±0.0120 |  | 0.0058±0.0063 | -0.0056±0.0093 |  | -0.0001±0.0110 | 0.0047±0.0119 |
| *Taipei cohort: Intervention group < Control group* | | | | |  |  |  |  |  |  |  |  |  |
| -38, -62, -12 | 1434 | 4.00 | <0.001 | Left temporal occipital fusiform cortex |  | -0.0035±0.0048 | -0.0012±0.0067 |  | -0.0015±0.0047 | -0.0032±0.0079 |  | -0.0044±0.0047 | 0.001±0.0057 |
